# Comparison of Topical 20% Azelaic Acid and 7.5% Dapsone in the Treatment of Mild‐To‐Moderate Papulopustular Rosacea

**DOI:** 10.1111/jocd.70212

**Published:** 2025-04-30

**Authors:** Sabir Hasanbeyzade

**Affiliations:** ^1^ Faculty of Medicine, Dr. Rıdvan Ege Research and Practice Hospital Ufuk University Ankara Türkiye; ^2^ Dermatology and Venerology Department Hitit University Erol Olcok Training and Research Hospital Corum Turkey

**Keywords:** azelaic acid, dapsone, rosacea

## Abstract

**Background:**

Rosacea is a chronic inflammatory skin disease, commonly affecting the central part of the face, characterized by erythema, flushing, telangiectasias, papules, and pustules. It may also involve sensations of burning, tingling, and occasionally fibrous changes.

**Aim:**

This study aims to compare the efficacy, side effect profiles, and patient satisfaction of topical 20% azelaic acid and 7.5% dapsone in the treatment of mild‐to‐moderate papulopustular rosacea (Stage 2).

**Methods:**

Ethics approval was obtained. A retrospective analysis was conducted on the medical records of 76 patients, including 44 in the azelaic acid group and 32 in the dapsone group, all diagnosed with mild‐to‐moderate papulopustular rosacea. These patients were treated with either topical azelaic acid or dapsone at the dermatology outpatient clinic between August 1, 2022 and December 31, 2022. Demographic characteristics, Investigator's Global Assessment scores, lesion counts, erythema scores, side effects, and patient satisfaction data were analyzed in the study.

**Results:**

No statistically significant differences were found between groups based on pretreatment IGA values, separate IGA scores (2–4), lesion counts, average erythema scores, or separate erythema scores (*p* > 0.05 for all). Within each group, comparisons of pre‐ and posttreatment IGA values, lesion counts, and erythema scores revealed statistically significant differences (*p* < 0.001 for all), indicating that both treatments were effective. When comparing the groups based on posttreatment IGA values, separate IGA scores (0–3), improvement percentages in IGA values, lesion counts, improvement percentages in lesion counts, average erythema scores, separate erythema scores (0–2), and improvement percentages in erythema scores, no significant differences were observed (*p* > 0.05 for all).

**Conclusion:**

Topical dapsone is as effective as azelaic acid in treating mild‐to‐moderate papulopustular rosacea and is associated with fewer side effects, making it a safer option.

## 1 Introduction

Rosacea is a chronic, recurrent inflammatory skin disease that typically affects the central part of the face, characterized by erythema, flushing, telangiectasias, papules, and pustules. The condition may also present with sensations of burning, tingling, and pricking, as well as, in some cases, fibrous changes [1]. It is commonly seen between the ages of 30 and 60 [2, 3] and is three times more common in women than in men [4]. The exact pathogenesis of rosacea remains unclear, but its pathology involves dilation of dermal vascular structures, signs of inflammation, and fibrotic changes [5]. The primary triggering factor is thought to be oxidative stress caused by free radicals, such as reactive oxygen species, released from inflammatory cells, especially neutrophils, which lead to damage in the perivascular and vascular collagen and elastin fibers [6, 7, 8]. In the current classification, rosacea is divided into four subtypes: erythematotelangiectatic rosacea, papulopustular rosacea, phymatous rosacea, and ocular rosacea [1]. There is also a granulomatous variant. Multiple subtypes can be observed simultaneously in a single patient [1].

In this retrospective study, we compared the efficacy, side effect profiles, and patient satisfaction levels of topical 20% azelaic acid (AA) and 7.5% dapsone (D) in mild‐to‐moderate papulopustular rosacea (Stage 2) patients (AA: Azelderm 20% cream, license holder: ORVA İlaç San. ve Tic. A.Ş., Atatürk Organize Sanayi Bölgesi, Çiğli/İzmir/Türkiye and D: Vulgarex 7.5% gel, license holder: Farma‐Tek İlaç Sanayi ve Ticaret A.Ş., Ümraniye/Istanbul/Türkiye).

## 2 Materials and Methods

### 2.1 Ethics Statement

Ethics approval for this study was obtained from the noninterventional ethics committee.

### 2.2 Study Design

This is a retrospective study. The medical records of patients who presented to the Dermatology outpatient clinic between August 1, 2022 and December 31, 2022 and were diagnosed with Stage 2 papulopustular rosacea were retrospectively reviewed.

### 2.3 Inclusion Criteria

Patients who were diagnosed with Stage 2 papulopustular rosacea, with mild‐to‐moderate rosacea (IGA scores ranging from 2 to 4) according to the Investigator's Global Assessment (IGA) [9] (Table 1), treated with either 20% AA or 7.5% D, and who were followed for at least 12 weeks in the hospital records, were included in the study.

**TABLE 1:** Investigator's Global Assessment: 7‐point score.

Score	Definition	Description
0	Clear	No inflammatory lesions, no or residual erythema
1	Minimal/almost clear	Rare papules and/or pustules, residual, or mild erythema
2	Mild	Few papules and/or pustules, mild erythema
3	Mild to moderate	Distinct number of papules and/or pustules, mild to moderate erythema
4	Moderate	Pronounced number of papules and/or pustules, moderate erythema
5	Moderate to severe	Many papules and/or pustules with large inflamed lesions, moderate erythema
6	Severe	Numerous papules and/or pustules, with confluent areas of inflamed lesions, moderate to severe erythema

### 2.4 Exclusion Criteria

Patients who were using any medications other than the aforementioned treatments at the time of rosacea diagnosis, those with ocular involvement, severe or very severe rosacea, steroid‐induced rosacea, those who had used topical steroids, antibiotics, or retinoids on the facial area in the last month, systemic steroids or antibiotics within the last month, or systemic isotretinoin treatment within the last 6 months, either for rosacea or any other condition, were excluded from the study.

At the end, a total of 76 patient records were included in the study: 44 patients in the AA group and 32 patients in the dapsone group. Data were collected from patient medical records, including pre‐ and posttreatment IGA scores, lesion counts, erythema scores (Table 2), side effects, and patient satisfaction levels. Improvement percentages in IGA scores, erythema scores, and lesion counts were also calculated. All patients applied their medications in the evening before bedtime and washed their faces upon waking up in the morning. Since erythema and scaling are common components of rosacea, any exacerbations of these conditions were recorded as side effects of the treatment.

**TABLE 2:** Erythema severity score.

Score	Rating	Description
0	None	No visible erythema
1	Mild	Slight erythema
2	Moderate	Pronounced erythema
3	Severe	Severe erythema with a red to purple hue

### 2.5 Statistics

The data collected for the study were analyzed using SPSS Statistics 25 (IBM, USA) software. Continuous variables were expressed as means and standard deviations (SD), whereas categorical variables were presented as frequencies and percentages. The comparison of categorical variables between the groups was conducted using Chi‐square tests. The Kolmogorov–Smirnov test was used to evaluate the normal distribution of continuous variables. For continuous variables that did not follow a normal distribution (pre‐ and posttreatment IGA scores, lesion counts, and erythema scores), the Mann–Whitney *U* test was used for comparisons between the two groups. Statistical significance was set at an alpha error of less than 5%.

## 3 Results

A total of 44 patients using AA with a mean age of 40.32 ± 5.13, and 41 of whom were female (93.2%), and 32 patients using D with a mean age of 39.69 ± 5.70, and 30 of whom were female (93.8%), were included in the study. No statistically significant difference was found between the groups in terms of gender and age (*p* > 0.05 for both). The demographic characteristics are presented in Table 3.

**TABLE 3:** Demographic characteristics, pre‐ and posttreatment IGA values, lesion numbers, erythema scores, and their improvement percentages.

Mean ± SD	Azelaic acid (*n* = 44)	Dapsone (*n* = 32)	*p*
Age (years)	40.32 ± 5.13	39.69 ± 5.70	0.692
Gender (female, *n*/%)	41/93.2%	30/93.8%	0.921
*Initial IGA values*	3.43 ± 0.70	3.50 ± 0.72	0.583
2 (*n*/%)	5/11.4%	4/12.5%	0.695
3	15/34.1%	8/25%	
4	24/54.5%	20/62.5%	
*EOT IGA values*	1.20 ± 0.88	1.41 ± 0.91	0.356
0 (*n*/%)	10/22.7%	5/15.6%	0.767
1	18/40.9%	13/40.6%	
2	13/29.5%	10/31.3%	
3	3/6.8%	4/12.5%	
IGA improvement percentage	67.61 ± 22.75	61.98 ± 23.47	0.341
Clear/almost clear (*n*/%)	28/63.6%	18/56.3%	0.515
≥ 2 IGA improvement (*n*/%)	39/88.6%	25/78.1%	0.215
Inıtıal NIL	20.95 ± 3.35	20.91 ± 3.69	0.962
EOT NIL	8.91 ± 5.61	10.16 ± 5.86	0.458
NIL improvement percentage	59.62 ± 24.98	53.05 ± 26.41	0.237
*Initial erythema score*	1.77 ± 0.60	1.84 ± 0.63	0.630
1 (*n*/%)	14/31.8%	9/28.1%	0.866
2	26/59.1%	19/59.4%	
3	4/9.1%	4/12.5%	
*EOT erythema score*	0.64 ± 0.57	0.88 ± 0.49	0.053
0 (*n*/%)	18/40.9%	6/18.8%	0.122
1	24/54.5%	24/75%	
2	2/4.5%	2/6.3%	
Erythema improvement percentage	64.77 ± 34.52	50.52 ± 32.92	0.079
≥ 1 erythema improvement (*n*/%)	38/86.4%	25/78.1%	0.346

Abbreviations: EOT, end of treatment; NIL, number of inflammatory lesions.

When comparing the groups based on pretreatment average IGA values, separate IGA scores (2–4), lesion counts, average erythema scores, and separate erythema scores (1–3), there were no notable differences between the groups regarding any variable (*p* > 0.05 for all, Table 3).

Within each group, comparisons of pre‐ and posttreatment IGA values, lesion counts, and erythema scores revealed statistically significant differences (*p* < 0.001 for all), indicating that both treatments were effective (Table 3).

**FIGURE 1:**
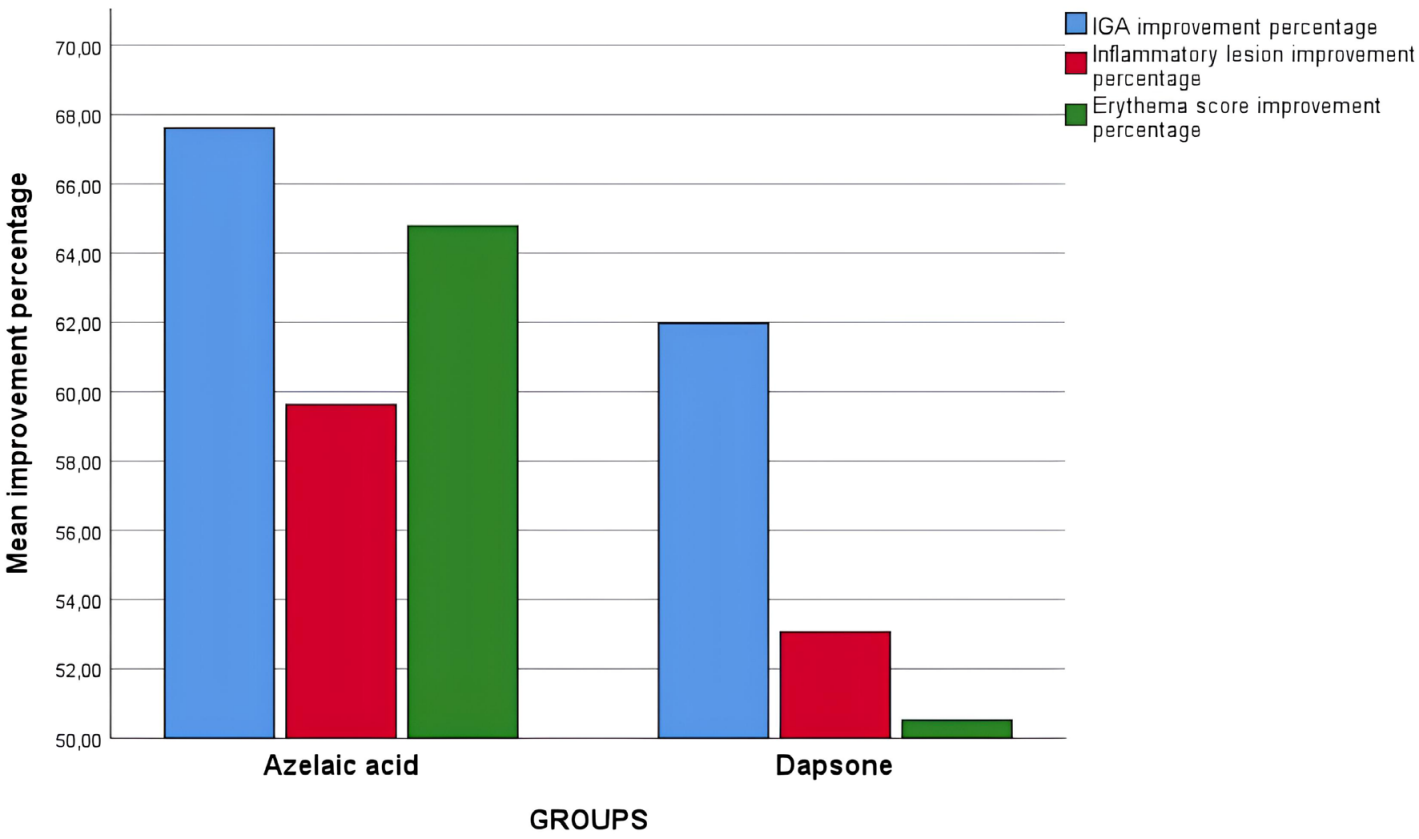
IGA, lesion number, and erythema score improvement percentages according to groups.

Subsequently, the groups were compared in terms of posttreatment average IGA values, separate IGA scores (0–3), improvement percentages in IGA values, lesion counts, improvement percentages in lesion counts, average erythema scores, separate erythema scores (0–2), and percentage of improvement in erythema scores. No significant differences were observed between the groups for any of the variables (*p* > 0.05 for all, Table 3, Figure 1).

Then, all patients were divided into “those with improvement of 2 or more” and “others” according to their posttreatment IGA values, as well as “those with 0‐1 IGA scores” (complete responders) and “others”, and the groups were compared in terms of these variables. No significant differences were found between the groups regarding any of the variables (*p* > 0.05 for both). Finally, all participants were divided into two as “those with improvement of 1 or more” and “others” according to their posttreatment erythema scores, and no significant differences were found between the groups in this regard (*p* > 0.05). Details are summarized in Table 3.

Patients with pretreatment IGA scores of 2, 3, and 4 were analyzed separately in order to compare the effects of the treatments. Since all patients in the AA and D groups with a pretreatment IGA score of 2 (5 patients in the AA group and 4 patients in the D group) showed “complete resolution”, statistical analysis could not be performed. Except for this, no significant differences were found for any variable in any group, indicating that the effectiveness of the treatments was similar. Detailed data are presented in Table 4.

**TABLE 4:** Recovery percentages in groups separated according to pretreatment IGA values.

Mean ± ss	2			3			4		
	AA (*n* = 5)	D (*n* = 4)	*p*	AA (*n* = 15)	D (*n* = 8)	*p*	AA (*n* = 24)	D (*n* = 20)	*p*
IGA improvement percentage	100	87.5 ± 25	0.264	73.3 ± 22.5	66.7 ± 25.2	0.523	57.3 ± 17.3	55 ± 19.2	0.720
≥ 2 IGA improvement (*n*/%)	5/100%	3/75%	0.236	13/86.7%	6/75%	0.482	21/87.5%	16/80%	0.498
Clear/almost clear (*n*/%)	5/100%	4/100%	X	13/86.7%	6/75%	0.482	10/41.7%	8/40%	0.911
Improvement percentages in lesion counts	100	81.3 ± 37.5	0.264	65.6 ± 28.7	55.9 ± 29.4	0.451	48.1 ± 12.6	46.3 ± 19.4	0.841
Erythema improvement percentage	100	75 ± 50	0.264	63.3 ± 44.2	37.5 ± 44.3	0.188	58.3 ± 26.9	50.8 ± 21.3	0.426
≥ 1 erythema improvement (*n*/%)	5/100%	3/75%	0.236	11/73.3%	4/50%	0.263	22/91.7%	18/90%	0.848

Abbreviations: AA, azelaic acid; D, dapsone.

When the groups were compared in terms of the presence or absence of side effects, the AA group showed a significantly higher frequency of side effects (*p* = 0.032). Upon reviewing the records of patients who experienced side effects, two types of side effects were observed (erythema and a burning sensation or dryness and flaking). Although erythema and burning sensations were more common in the AA group, no statistically significant differences were found between the groups. Details are presented in Table 5.

**TABLE 5:** Side effects seen in patients.

*n*/%	Azelaic acid (*n* = 44)	Dapsone (*n* = 32)	*p*
No	29/65.9%	28/87.5%	0.032
Yes	15/34.1%	4/12.5%	
Erythema/burning	12/80%	2/50%	0.226
Flaking/desquamation	3/20%	2/50%	

Although a higher percentage of patients in the AA group reported being satisfied/very satisfied, and a higher percentage in the D group reported being dissatisfied, no significant difference was found between the groups in terms of patient satisfaction. Details are summarized in Table 6.

**TABLE 6:** Satisfaction rates of patients.

*n*/%	Azelaic acid (*n* = 44)	Dapsone (*n* = 32)	*p*
Very satisfied	12/27.3%	8/25%	0.805
Satisfied	16/36.4%	10/31.3%	
Dissatisfied	16/36.4%	14/43.8%	

When each group was compared separately within itself based on pretreatment IGA values and posttreatment patient satisfaction, it was observed that in both groups, patients with a pretreatment IGA value of 4 had higher rates of dissatisfaction following treatment (*p* = 0.012 and 0.002, respectively).

## 4 Discussion

AA, also known as 1,7‐heptanedicarboxylic acid, is a medium‐chain saturated dicarboxylic acid. AA competitively inhibits tyrosinase and mitochondrial oxidoreductases, as well as some enzymes involved in DNA synthesis [10, 11]. The use of 20% AA in rosacea was first reported in 1991 [12]. AA has antibacterial, anti‐keratinizing, and anti‐inflammatory properties. By inhibiting free radical release from neutrophils, it also prevents oxidative stress‐induced damage [13, 14].

Dapsone (4‐amino‐4‐diphenyl sulfone) is a sulfon group drug that was discovered in 1908 [15]. It exerts its effects by competitively inhibiting dihydropteroate synthase, thereby suppressing dihydrofolate synthesis. Dapsone also demonstrates antibacterial activity against Cutibacterium acnes [16], inhibits neutrophil chemotaxis [17], and prevents myeloperoxidase‐mediated damage [18].

Rosacea is approximately three times more common in women than in men. However, in our study, 93%–94% of all patients were women. We hypothesize that women may be more concerned with their appearance, which could contribute to a higher rate of hospital visits. The disease is typically more prevalent in individuals aged 30–60 years, and the average age of patients in our study was around 40.

Faghihi et al. conducted a double‐blind, randomized controlled trial with 56 patients to compare the efficacy of 5% topical dapsone and 0.75% topical metronidazole in the treatment of papulopustular rosacea. In the dapsone group, the lesion count was 15 ± 7.4 before treatment and reduced to 11.1 ± 6 after treatment (*p* < 0.0001), indicating that dapsone was as effective as metronidazole [19]. Similarly, in our study, we found a significant difference in the lesion count before and after treatment.

Carmichael et al. [20] conducted a split‐face and vehicle‐controlled study on 33 rosacea patients with at least 10 papules and/or pustules. After 9 weeks of treatment, the side treated with 20% AA cream showed an 82% reduction in inflammatory lesions, whereas the vehicle‐treated side showed a 56% improvement (*p* < 0.0001). In our study, we found a 60% reduction in inflammatory lesions in the group treated with AA.

Bjerke et al. conducted a parallel‐group, vehicle‐controlled study on 116 rosacea patients with at least 10 papules and/or pustules. At the end of 3 months of treatment, there was a 73% decrease in inflammatory lesions in the group using 20% AA, whereas a 51% decrease was seen in the vehicle group, and this difference between the groups was statistically significant [21]. In our study, the improvement rate was slightly lower (60%).

Maddin et al. conducted a split‐face comparative study with 40 rosacea patients who had at least 10 papules and/or pustules. In this study, the group using 20% AA twice daily for 15 weeks showed a 79% reduction in inflammatory lesions, whereas the group using 0.75% metronidazole showed a 69% reduction. However, this difference was not statistically significant [22]. In our study, the reduction in inflammatory lesions in the AA group was 60%. In their study, the AA group showed a 26% reduction in erythema, whereas the other group showed a 19% reduction, but again, the difference was not statistically significant. In contrast, we found a higher percentage of improvement in erythema scores in the AA group in our study, with a reduction of 65%.

Elewski et al. [9] conducted a multicenter, double‐blind, randomized, parallel‐group study involving a total of 251 rosacea patients with 10–50 papules and/or pustules. The study compared two groups: one applying 15% AA twice daily (124 patients) and the other applying 0.75% metronidazole twice daily (127 patients) for 15 weeks. They found that the AA group had a significantly greater reduction in the number of inflammatory lesions compared to the metronidazole group (72.7% vs. 55.8%, *p* < 0.001). Our study showed similar results, with a 60% reduction in the AA group.

Özkoca et al. conducted an 8‐week prospective study with 32 papulopustular rosacea patients to investigate the efficacy and side effect profile of topical 7.5% dapsone applied in the evening [23]. At the end of the treatment, the average lesion counts were 3.87 ± 3.76 and the average IGA scores were 0.74 ± 0.73. They observed statistically significant differences in pre‐ and posttreatment lesion counts and IGA scores (*p* < 0.001 for both). No side effects were reported during the treatment.

## 5 Conclusion

AA has been used topically in the treatment of rosacea for approximately 35 years. In contrast, the use of topical dapsone in rosacea is less common. In this study, no statistically significant differences were observed between topical 7.5% dapsone and topical 20% AA applied once daily in the evening for the treatment of mild‐to‐moderate papulopustular rosacea (Stage 2) according to the efficacy and patient satisfaction. Consequently, topical 7.5% dapsone, when applied once daily in the evening, demonstrates comparable efficacy to topical 20% AA in managing rosacea. Notably, the dapsone group exhibited fewer side effects, whereas erythema and a burning sensation were more commonly reported in the AA group. These findings suggest that topical 7.5% dapsone may be a safer alternative to AA for the treatment of papulopustular rosacea.

Therefore, topical dapsone is as effective as topical AA in the treatment of Stage 2 mild‐to‐moderate papulopustular rosacea and is safer in terms of side effects. However, in order to further clarify this issue, split‐face or controlled studies with a larger number of patients are needed.

### 5.1 Limitations

One of the shortcomings of our study is its retrospective design. Additionally, since our study was conducted on patients who visited a certain hospital in a particular region and due to the small sample size, prospective, multicenter, case‐controlled studies with larger patient numbers are needed in order for the results to be generalized to the population.

## Ethics Statement

The study was approved by the clinical trials ethics committee of Hitit University Erol Olcok Training and Research Hospital (approval date 16.03.2023 and number 2023‐25).

## Conflicts of Interest

The author declares no conflicts of interest.

## Data Availability

The data that support the findings of this study are available from the corresponding author upon reasonable request.
